# Supplementary material for: Real-time finite element analysis allows homogenization of tissue scale strains and reduces variance in a mouse defect healing model
Source: Sci Rep. 2021 Jun 29;11:13511. doi: 10.1038/s41598-021-92961-y (PMC8241979; doi:10.1038/s41598-021-92961-y)
Supplement: Supplementary file 1 — Supplementary Figure Legend. [file 41598_2021_92961_MOESM1_ESM.docx]

**Supplementary Figure S1:** Kolmogorov-Smirnov tests indicate that after a week of adaptive loading, the strain distributions of loaded mice similar. However once adaptive loading stops, and a non-adaptive constant load is applied the difference in distribution increase rapidly. Adaptive loading also arrested the increase in dissimilarity between the control and loading group.
