# Supplementary figures and images for: Real-time finite element analysis allows homogenization of tissue scale strains and reduces variance in a mouse defect healing model
Source: Sci Rep. 2021 Jun 29;11:13511. doi: 10.1038/s41598-021-92961-y (PMC8241979; doi:10.1038/s41598-021-92961-y)

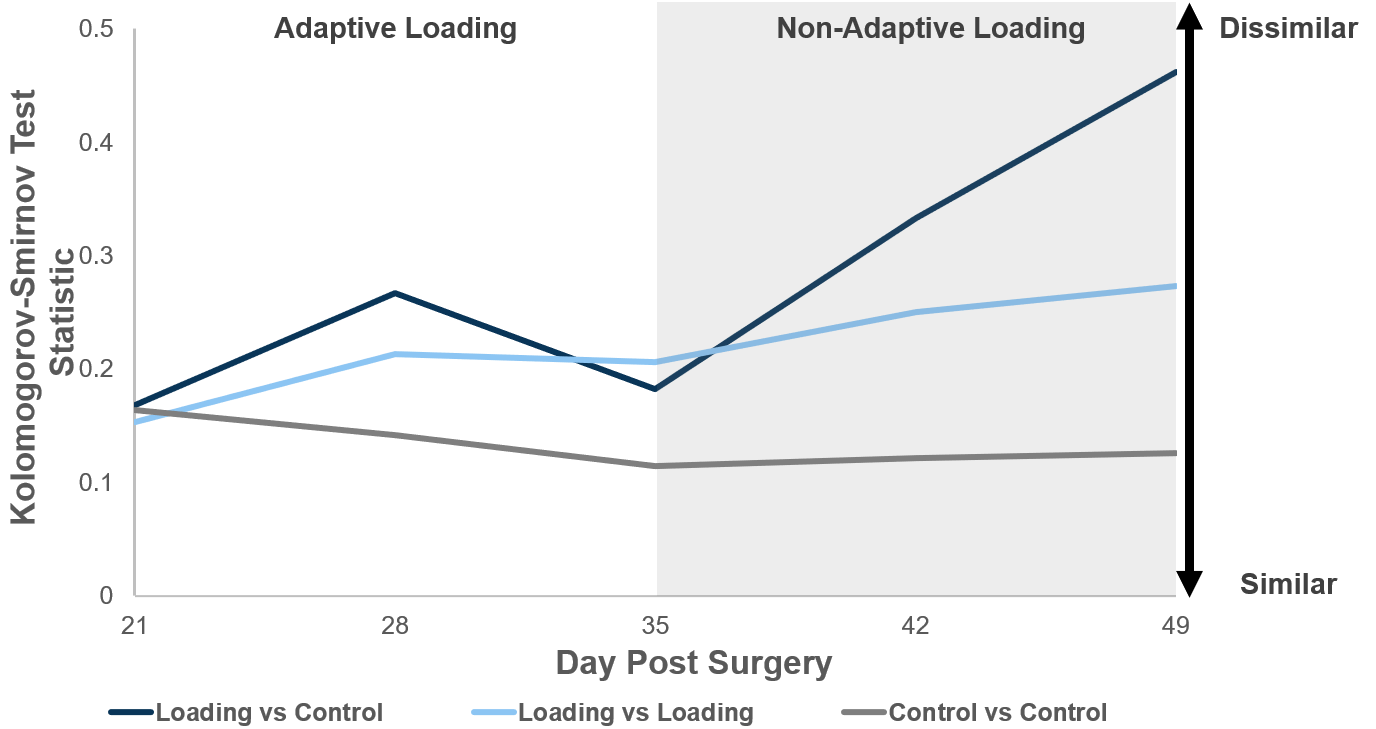

Supplement: Supplementary file 2 — Supplementary Figure S1. [file 41598_2021_92961_MOESM2_ESM.png]
